# A theoretical model for the development of a diagnosis-based clinical decision rule for the management of patients with spinal pain

**DOI:** 10.1186/1471-2474-8-75

**Published:** 2007-08-03

**Authors:** Donald R Murphy, Eric L Hurwitz

**Affiliations:** 1 Rhode Island Spine Center, Pawtucket, RI USA; 2 Department of Community Health, Warren Alpert Medical School of Brown University, Providence, RI USA; 3 Research Department, New York Chiropractic College, Seneca Falls, NY USA; 4 Department of Public Health Sciences, John A. Burns School of Medicine, University of Hawaii at Mânoa, Honolulu, Hawaii USA

## Abstract

**Background:**

Spinal pain is a common problem, and disability related to spinal pain has great consequence in terms of human suffering, medical costs and costs to society. The traditional approach to the non-surgical management of patients with spinal pain, as well as to research in spinal pain, has been such that the type of treatment any given patient receives is determined more by what type of practitioner he or she sees, rather than by diagnosis. Furthermore, determination of treatment depends more on the type of practitioner than by the needs of the patient. Much needed is an approach to clinical management and research that allows clinicians to base treatment decisions on a reliable and valid diagnostic strategy leading to treatment choices that result in demonstrable outcomes in terms of pain relief and functional improvement. The challenges of diagnosis in patients with spinal pain, however, are that spinal pain is often multifactorial, the factors involved are wide ranging, and for most of these factors there exist no definitive objective tests.

**Discussion:**

The theoretical model of a diagnosis-based clinical decision rule has been developed that may provide clinicians with an approach to non-surgical spine pain patients that allows for specific treatment decisions based on a specific diagnosis. This is not a classification scheme, but a thought process that attempts to identify most important features present in each individual patient. Presented here is a description of the proposed approach, in which reliable and valid assessment procedures are used to arrive at a working diagnosis which considers the disparate factors contributing to spinal pain. Treatment decisions are based on the diagnosis and the outcome of treatment can be measured.

**Summary:**

In this paper, the theoretical model of a proposed diagnosis-based clinical decision rule is presented. In a subsequent manuscript, the current evidence for the approach will be systematically reviewed, and we will present a research strategy required to fill in the gaps in the current evidence, as well as to investigate the decision rule as a whole.

## Background

Chronic spinal pain is an increasingly common problem in Western Society [1]. Spinal disorders exact great costs, in terms of both direct medical costs and indirect costs related to disability and lost productivity [1-3]. A number of researchers have attempted to improve our ability to identify the causes of spinal pain as well as to diagnose and treat patients with this problem. In spite of this, accurate diagnosis, leading to specific, targeted treatments, of patients with spinal pain has been elusive.

It has been repeated over the years that only in 15% of patients with spinal pain can a definitive diagnosis be made [4-6]. However, if one surveys the spine literature, one finds a variety of methods for detecting many of the factors that are believed to be of importance, most of which have known reliability and validity, although there are some that do not. Each of these methods may only help the clinician to identify one particular potential contributing factor in the overall clinical picture of the spine pain patient. However, it may be possible that, by utilizing many of the various diagnostic procedures available to the spine clinician, one can develop a specific working diagnosis that encompasses all of the dimensions for which there may be contributing factors and from which a management strategy may be designed that addresses each of the most important factors in each individual patient.

The purpose of this paper is to present the theoretical model of a diagnosis-based clinical decision rule (DBCDR) for the diagnosis and non-surgical management of patients with spinal pain. The model considers a number of known or suspected factors that contribute to the clinical picture and allows for the development of a management strategy that is derived from the multifactorial diagnosis. This paper presents the conceptual model of this clinical decision rule and its application in the clinical setting. In a subsequent manuscript we will systematically review the evidence regarding the components of the approach, and present those areas of research needed to investigate its validity and usefulness to spine clinicians.

## Discussion

### The three essential questions of diagnosis

The DBCDR is based on what the authors refer to as the 3 essential questions of diagnosis. It is suggested here that the answers to these questions supply the clinician with the most important information required to develop a specific diagnosis from which a management strategy can be derived. The 3 questions are:

### The first question of diagnosis: Are the patient's symptoms reflective of a visceral disorder or a serious or potentially life-threatening illness?

There are several diseases for which spinal pain may be among the initial symptoms. It is important for any physician seeing patients with spinal pain to be aware of these disorders and to be skilled enough in differential diagnosis to detect, or at least suspect, their presence. The most important diagnoses are listed in Table 1.

**Table 1:** Visceral or potentially serious or life threatening diseases that can present as spinal pain.

**Disorder**	**Detected by**
Cancer	Previous history of CA, no position of relief, fever, constitutional symptoms, weight loss
Benign tumor	Localized severe pain, no position of relief, dramatic relief with NSAID, pain on percussion
Infection	History of fever and/or chills, fever on examination, pinpoint tenderness, redness or heat
Fracture	History of trauma, history of osteoporosis, pain on percussion
Seronegative spondyloarthropathy	Hx of iritis, AM stiffness, improvement with exercise, family Hx
GI disease	GI complaints, relation of pain to certain foods, abdominal examination
GU disease	GU complaints, bleeding, spotting, unusual discharge, GU examination
Myelopathy	Gait difficulties, bowel/bladder dysfunction, UMN signs, spasticity, sensory level
Cauda equina syndrome	Bowel/bladder difficulties, saddle anesthesia, decreased anal sphincter tone

CA – cancer; NSAID – non-steroidal anti-inflammatory drugs; Hx – history; AM – morning; GI – gastrointestinal; GU – genitourinary; UMN – upper motor neuron

### The second question of diagnosis: From where is the patient's pain arising?

In asking this question, the clinician is not necessarily attempting to determine the precise tissue of origin, but rather is trying to identify characteristics about the pain source that allow treatment decisions to be made. There are a number of tissues in the spine that have the potential to generate pain. However, while a few studies have suggested that clinical factors can be used to detect specific pain generators in some instances [7,8], to a large extent, methods that allow clinicians to make an unequivocal tissue-specific diagnosis have been elusive. There is good evidence, however, that historical factors and examination procedures can allow one to identify certain characteristics in each individual patient that may be useful in making treatment decisions. Proposed here are 4 signs of greatest importance in seeking the answer to the second question of diagnosis (Table 2). A variety of clinical tests have been developed that allow the clinician to attempt to identify the origin of the patient's pain. Part 2 of this paper reviews the reliability and validity of these clinical procedures.

**Table 2:** Proposed pain provocation signs and their means of detection.

Pain Provocation Sign	Detection	Suspected Source
Segmental Pain Provocation Signs	Palpation, pain provocation tests	Zygapophyseal joint
Centralization Signs	End range loading examination	Intervertebral disc
Neurodynamic Signs	Neurodynamic Tests	Neural structures
Muscle Palpation Signs	Palpation	Myofascial tissues

#### Centralization signs

Centralization signs are detected through methods originally developed by McKenzie [9,10]. The examination procedure involves moving the spine to end range in various directions and monitoring the mechanical and symptomatic response to these movements. Traditionally, the findings of this examination have been thought to identify the intervertebral disc as the source of pain, and some experimental evidence supports this [11-13], though further work in this area is needed. Nonetheless, the centralization sign has been demonstrated to be useful in prognosis [14] and in helping the clinician to make decisions regarding the best form of treatment for this particular aspect of the clinical picture [15].

#### Segmental pain provocation signs

Examination for pain provocation signs involves the clinician attempting to reproduce the patient's pain by apply maneuvers designed to stress segmental tissues. In the cervical, thoracic and lumbar spine, this involves segmental palpation. This palpation is designed to assess for pain response, not necessarily movement abnormality [16-25]. In the sacroiliac area, pain provocation tests are used [8,13,26], and the usefulness of these tests is enhanced when they are performed in conjunction with examination for centralization signs [27] (see below). Some evidence suggests that these signs are reflective of pain arising from joint structures [8,24,28]. However, as will be seen with the other signs discussed here, it is interesting, and sometimes useful, to speculate about the precise pain generating tissue responsible for producing pain with segmental palpation, but it is not necessary to know the precise pain generating tissue in making treatment decisions based on the identification of these signs.

#### Neurodynamic signs

Neurodynamic signs involve the reproduction of pain resulting from tests designed to apply stress to neural structures. In the spine, neurodynamic signs most likely arise from radicular pain, most commonly arising from lateral canal stenosis and disc protrusion [29,30]. Neurodynamic signs are derived from clinical neurodynamic tests [31] and other pain provoking procedures [32]. These can be supported with historical factors as well as neurologic examination [7].

#### Muscle palpation signs

Muscle palpation signs involve the reproduction of pain from direct palpation of muscles. These signs have typically been thought to implicate the presence of myofascial trigger points (TrPs) [33]. Again, as with other pain generating signs, knowing the precise mechanism is not absolutely necessary in order to make diagnostic and treatment decisions.

Pain referral pattern maps from various muscles have been developed [33] and are in popular use, although only a few of these have been investigated for validity [34,35]. Knowledge of these referred pain patterns, and comparing them with the pain pattern described by the patient, may allow the clinician to make decisions regarding which muscles should be examined with palpation.

### The third question of diagnosis: What has gone wrong with this person as a whole that would cause the pain experience to develop and persist?

With this question, the clinician attempts to determine if there are any factors other than the pain generating tissue that serves to maintain or perpetuate the pain experience. It would appear that an essential aspect to effective management of patients with spinal pain would include the identification and management of those factors that place the acute or subacute spinal pain patient at risk of developing ongoing problems or, in the case of the chronic or recurrent spinal pain patient, that contribute to the perpetuation of pain and dysfunction. Table 3 lists the factors believed to be of greatest importance according to current evidence. These factors encompass biomechanical, neurophysiological and psychological processes.

**Table 3:** Factors presumed to be of greatest importance in the perpetuation of spinal pain.

Dynamic Instability (impaired motor control)	Fear
Oculomotor dysfunction	Catastrophizing
Central pain hypersensitivity	Passive coping
	Depression

#### Dynamic instability (impaired motor control)

Several studies have demonstrated alteration in motor control strategies in patients with spinal pain [36-38]. It is believed that this altered motor control leads to decreased stability of the spine [39-41], thus predisposing the spine to injury and perpetuating chronic spinal pain. Several clinical examination procedures have been suggested to identify impaired motor control in patients with pain in the cervical spine [36,42-46], the lumbar spine [47,48] pelvis [49].

#### Central pain hypersensitivity (CPH)

CPH is a state in which an alteration has occurred in the manner in which nociceptive information is received, processed and modulated, which serves to heighten the pain experience [50-53]. Increased pain sensitivity and decreased pain thresholds have been found in patients with chronic neck pain [54-56], chronic low back pain (LBP) [57] and chronic headache [58].

The examination for Waddell's nonorganic signs [59] is a popular procedure that was developed for the purpose of identifying a behavioral component of the clinical picture in patients with LBP. A recent systematic review of the literature by Fishbain, et al [60] found good evidence to suggest that these signs are associated with heightened pain perception.

#### Oculomotor dysfunction

Many studies have found impairment of oculomotor reflexes in patients with chronic neck pain after trauma [61-67] and in patients with chronic tension type headache [68]. Also, treatments aimed at improving oculomotor function have been found to be useful in decreasing neck pain and neck pain-related disability [69,70].

Simple and practical tests for oculomotor reflex function for use in the typical clinical setting are nonexistent. However, Heikklla and Wenngren [63] found significant correlation between the finding of poor performance on oculomotor tests and a test for head repositioning accuracy. Head repositioning accuracy can be measured in the clinic [71].

#### Fear and catastrophizing

Both fear and catastrophizing have been shown to be important predictors of present pain intensity and disability [72,73] and of future chronicity [74-77]. Several instruments have been validated for measuring these factors [73,78-80].

#### Passive coping

As with fear and catastrophizing, passive coping has been shown to contribute to present disability [81] and to predict future disability [82] and can be measured in the clinic [83].

#### Depression

As with the psychological factors discussed previously, depression has been shown to contribute to present disability [75,81] and predict future disability [84-86]. It can be measured via questionnaire [87].

### Arriving at a diagnosis and formulating a management strategy

#### Arriving at a diagnosis

**Figure 1:**
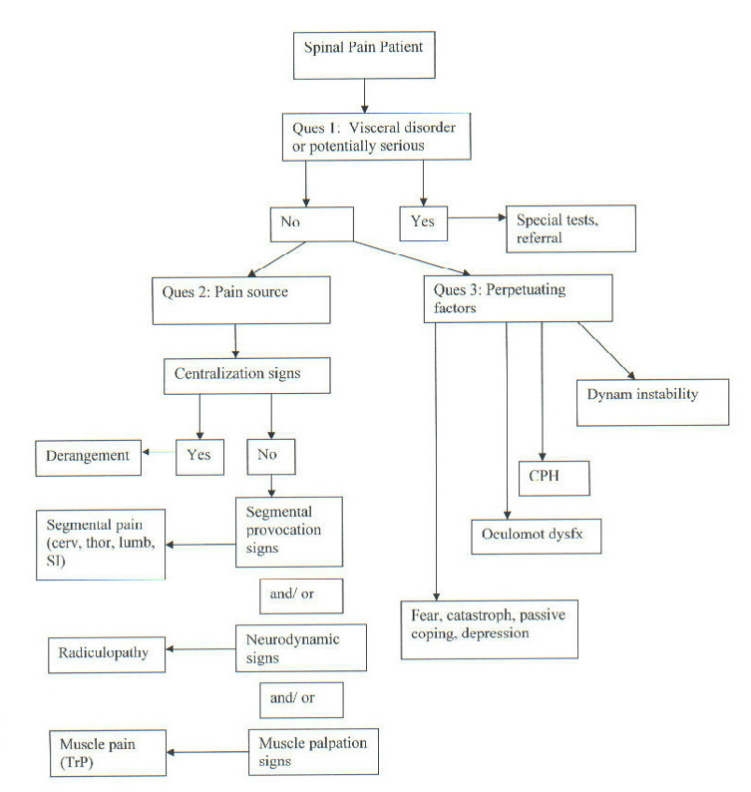
Diagnostic algorithm for the application of the DBCDR.

The DBCDR is not a classification method. It is a diagnostic method. However, the "diagnosis", using the DBCDR, is not a traditional diagnosis, in which a diagnostic label is given to a disease entity based on the unique pathophysiology of the particular entity. Rather, it is a collection of signs, sometimes single and sometimes multiple, from which the clinician can make treatment decisions. Also, because of the absence of definitive findings on tests such as imaging in the majority of spinal pain patients, the diagnosis using the DBCDR is a working hypothesis that is tested through treatment. (Figure 1)

Some examples of diagnoses using the DBCDR might be:

1. Segmental pain provocation signs (question #2) and significant fear beliefs (question #3); rule out infection (question #1).

2. Centralization signs (question #2) with impaired motor control and CPH (question #3).

3. Segmental pain provocation signs with neurodynamic signs (question #2) and fear and catastrophizing (question #3); rule out myelopathy (question #1).

#### Formulating a management strategy

**Figure 2:**
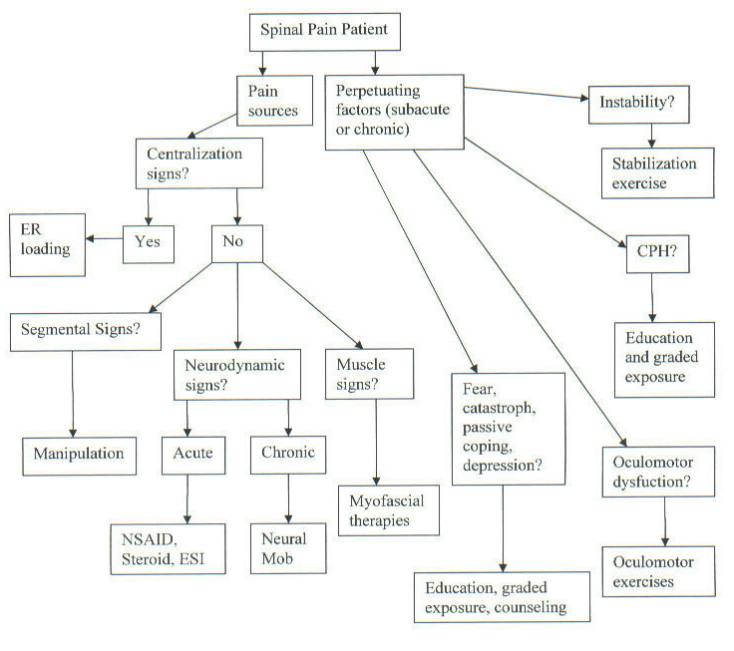
Management algorithm for the application of the DBCDR.

Once the clinician has established a working diagnosis (based on the answers to the 3 questions), a management strategy would be developed. An important concept of management in this model is that none of the important factors that may be present in any given spinal pain patient occurs in isolation. Pain generators and perpetuating factors interact in producing the clinical picture that practitioners see. (Figure 2)

In the model presented here, these are the management decisions that the clinician might make based on the findings of the diagnostic process:

### Management strategy in response to Question #1

Any significant findings suggestive of visceral disease or serious or potentially life threatening illness would be investigated with further diagnostic work up and/or referral.

### Management strategy in response to Question #2

#### Centralization signs

The recommended treatment of choice for centralization signs is end range loading maneuvers in the direction of centralization [15], which is part of the McKenzie method [10]. Because centralization signs can be addressed with exercises and self-care strategies that patients can apply themselves, these signs are always addressed first, regardless of the presence of other signs.

The DBCDR does not depend on a known tissue of origin of the patient's pain; however, as centralization signs may be associated with disc pain in the lumbar spine [11], another treatment that may have potential in the presence of these signs is distraction manipulation [88], a type of manipulation that utilizes a special treatment table and which has been shown to reduce intradiscal pressure [89,90]. However, whether and to what extent distraction manipulation may add to self- or practitioner-applied end range loading maneuvers has not been investigated.

#### Segmental pain provocation signs

In the context of the DBCDR, it is recommended that in patients with segmental pain provocation signs as well as centralization signs, end range loading maneuvers be utilized first, with no action being taken regarding the segmental pain provocation signs until end range loading has been fully explored. In patients with segmental pain provocation signs who do not exhibit centralization signs, manipulation is the recommended treatment. The rationale for this is that it is known that manipulation has segmental effects, [91,92] and segmental hypomobility form a component of a prediction rule for those patients with LBP who are most likely to benefit from manipulation [93]. Because of this, it seems reasonable to consider manipulation as a treatment of choice in the presence of segmental pain signs, and to explore this from a research perspective.

Zygapophyseal [94] or sacroiliac joint [95] injection may also be helpful in patients with segmental pain provocation signs. Radiofrequency denervation may be a last-resort treatment for patients with segmental pain provocation signs[96].

#### Neurodynamic signs

In the acute stage, especially with disc protrusion, radicular pain is thought to be largely chemical, as a result of inflammation [97]. As such, anti-inflammatory measures would appear to be a useful first line approach. These can come in the form of general approaches such as non-steroidal anti-inflammatory medications (NSAIDs) or oral steroids [98,99]. Epidural steroid injection (ESI) is more specific to radiculopathy [100]; selective nerve root anesthetic block is also an option for radiculopathy patients [101,102]. An anti-inflammatory diet [103] may also be beneficial.

In chronic stages, many patients (one study of lumbar radiculopathy patients [104] suggested approximately 50%) will exhibit centralization signs, in which cases end range loading in the direction of centralization is the recommended first-line approach. In those who do not exhibit centralization signs, or who have residual radicular symptoms, a treatment approach that holds promise is neural mobilization [31] which attempts to mobilize the involved nerve root to improve its mechanics and decrease its sensitivity [105-107]. Distraction manipulation is another option in patients with lumbar radiculopathy [107,108].

#### Muscle palpation signs

A multitude of treatments has been recommended for pain apparently arising from muscle, ranging from manual techniques such as ischemic compression [109], to muscle lengthening techniques [110], to trigger point injections.

### Management strategy in response to Question #3

#### Dynamic instability (impaired motor control)

Many methods have been recommended to improve motor control in the spine, usually involving "stabilization exercises" [40,111] that may utilize simple floor exercises, or low-technology equipment such as gymnastic balls, or high-technology equipment. General fitness or strength training exercise may also be of benefit [112,113].

#### Central pain hypersensitivity

As CPH requires ongoing peripheral nociceptive input for its perpetuation [53], effectively treating sources of this nociceptive input (i.e., responding to questions #2), would appear to be useful in addressing CPH.

Also, as CPH is a state in which the nociceptive system has become, in effect, hypersensitive, it would appear that a useful treatment would involve a desensitization process. It may be possible to accomplish this through a graded exposure approach [114].

#### Oculomotor dysfunction

It appears that oculomotor dysfunction occurs particularly in patients with whiplash injury [61,69] and tension-type headache [68] and is treated with exercises designed to train eye-head-neck movements [115,116].

#### Fear, catastrophizing, passive coping and depression

Evidence suggests that fear and catastrophizing are directly related to CPH [117,118]. In addition, it appears that passive coping and depression are a part of an overall psychological response on the part of the patient that has a detrimental effect on their ability to recover and resume normal activities [75]. Thus, in the context of the DBCDR, the recommended response to this would be to educate the patient about the presence and nature of CPH. This may reduce fear and catastrophizing, and encourage the patient to take a more active approach to coping, which would, in turn, positively impact depression. This education must then be followed by the graded exposure approach discussed earlier. It should be noted that fear, catastrophizing, passive coping and depression often improve with purely somatic-based treatments, when these treatments are successful in reducing pain [14,119-121].

In some patients in which the fear, catastrophizing, passive coping and depression are not overcome with the interventions discussed above, [75], further intervention by a psychologist or psychiatrist may be necessary.

### A comparison of the DBCDR with "classification" schemes for patients with spinal pain

Classically, the diagnosis of spinal pain has revolved around attempting to make a tissue-specific diagnosis based on pathoanatomy. The assumption was that if someone's back or neck hurts, it is the job of the practitioner to identify the tissue that is painful and the pathoanatomic or pathophysiologic process that is producing pain. Once this tissue and this process are identified, the diagnosis can be made. Diagnostic methods such as radiographs, CT, MRI and EMG were developed for this purpose. This is the way diagnoses are made in other areas of medicine, such as with GI or cardiac disorders. However, as research attempted to investigate better ways to make this pathoanatomic diagnosis, it was found that 1) many pathoanatomic entities occur in the absence of spinal pain and 2) many people with spinal pain do not have identifiable pathoanatomic or pathophysiologic processes that would explain their pain.

This led to the categorization of patients into 2 groups – "specific" spinal pain, i.e., those in whom a reliable and valid pathoanatomic diagnosis can be made, and "non-specific", i.e., those in whom the source of pain could not be identified. In recent years, a response to this limited categorization has come in the form of attempting to identify "subgroups" of spinal pain patients. This has resulted in various "classification systems" that have attempted to identify common characteristics in groups of spinal pain patients in the hope that certain treatments can be targeted to certain classifications of patients.

The Quebec Task Force on Spinal Disorders [6] developed a classification system based on signs and symptoms, imaging findings and response to treatment. This system was found to have some utility with regard to surgical decision making [122], it was still limited with regard to helping clinicians make specific treatment decisions in the majority of patients.

One classification system is that of McKenzie [10]. In this classification, three "syndromes" are considered. The clinician attempts to identify in each patient which of these syndromes is present, so that treatment can be applied that is appropriate for that syndrome.

According to McKenzie, the largest of these is the "derangement syndrome". In this group of patients, end range loading maneuvers are used to identify a characteristic pattern of "centralization" of symptoms when loading maneuvers are applied in a certain direction, and "peripheralization" of symptoms when loading maneuvers are applied in another direction (typically the direction opposite of that which produced centralization).

The McKenzie system, at least as it applies to the derangement syndrome, has been found to be efficacious for those patients for whom it applies [15,123,124]. However, only a limited (though sizable) percentage of patients with LBP have derangement. It has been estimated that approximately 70% of patients with acute LBP and approximately 50% with chronic LBP centralize with end range loading maneuvers, thus being classified as "derangement" [125]. It is unknown what percentage of patients with acute and chronic neck pain and thoracic pain can be classified as having derangement. It is also unknown what percentage of spinal pain patients can be classified as having the dysfunction and postural syndromes, and, while the reliability of these classifications have been found to be good [126-128], the validity is unknown.

So it appears that the McKenzie classification is useful in identifying at least one aspect of a diagnosis in most spinal pain patients. However, half the chronic LBP patients and an unknown percentage of cervical and thoracic pain patients do not have derangements and require some other methods with which to make a diagnosis. Nonetheless, the McKenzie approach plays a key role in the DBCDR presented here.

Another classification system was initially developed by Delitto [129] and used historical factors, symptom behavior and clinical signs to categorize spinal pain patients. This system evolved into one in which patients with LBP are placed into one of four categories [130]:

1. Immobilization

2. Mobilization

3. Specific exercise

4. Traction

A similar classification system was developed for neck pain patients by Childs, et al [131]. In this system, patients are classified into 5 categories [131]:

1. Mobility

2. Centralization

3. Conditioning and increased exercise tolerance

4. Pain control

5. Reduce headache

So, with these classification schemes, patients are categorized into one of four or five groups, rather than into one of two groups (i.e., "specific" and "nonspecific"). Evidence suggests that this has been a significant step forward, as patients with LBP who are treated according to this classification scheme have better outcomes than patients who are treated according to the two-category scheme [130]. It is unknown how this classification scheme applies to the cervical spine.

While this classification is a significant advancement in that it attempts to identify those patients who are most likely to respond to specific treatments, it has its limitations. For example, patients with LBP are placed in the "mobilization" (or manipulation) category based on the following features [93]:

1. Symptom duration < 16 days

2. No symptoms distal to knee

3. < 19 on a Fear-Avoidance Beliefs Q

4. At least 1 hypomobile lumbar segment

5. At least 1 hip with > 35 degrees of internal rotation

In the classification system for the cervical spine, patients are placed in the "mobility" category based on these features:

1. Recent onset of symptoms

2. No radicular or referred symptoms into the upper quarter

3. Restricted range of motion (ROM)

4. No signs of nerve root compression or peripheralization of symptoms with ROM

Evidence suggests that there are many patients who respond positively to manipulation who do not fit into these groups. For example, patients with chronic LBP and neck pain often respond to manipulation [132], as do many with radiculopathy or pain below the knee [106,107,133].

The proposed DBCDR is different from these other systems in that it is not a classification system; there are no classifications in which patients are placed. Rather, those factors that are known or suspected to contribute to the clinical picture of spinal pain and all are considered in each patient, with the recognition that many patients have a variety of factors involved in his or her clinical picture, and thus defy easy classification. It also attempts to find those clinical features that play a role in each individual case, and apply treatments designed to address those features. Thus, the clinician in not limited to 3, 4 or 5 classifications, but is free to manage each patient according to those clinical features that are deemed most relevant in each case. Further research is needed to determine whether the DBCDR is truly a useful "rule" in helping clinicians make diagnostic and treatment decisions.

## Summary

The traditional management of patients with spine related disorders has typically been driven more by the training of the individual clinician than the needs of the patient. Research on treatments for spinal pain has placed patients into homogeneous groups, as if all patients had the same diagnosis. This has led to the conclusion among many that no treatment for spinal pain has a great deal to offer [5,134]. A novel approach to the diagnosis and management of patients with spine-related disorders is presented that may help to improve the precision of medical decision making and thus improve treatment outcome. It is based on what the authors refer to as the 3 essential questions of diagnosis.

The answers to these questions may allow the clinician to develop a working diagnosis from which a management strategy can be developed. This strategy is designed to address the most important factors suspected to be contributing in each case.

The theoretical model of this proposed diagnosis-based clinical decision rule is presented here. In a subsequent paper, the evidence as is currently exists related to this model will be systematically reviewed and a research strategy will be presented, the purpose of which is to determine whether the model has sufficient reliability, validity and efficacy to recommend it as an alternative approach to patients with spinal pain.

## Competing interests

The author(s) declare that they have no competing interests.

## Authors' contributions

DRM conceived of the idea of the diagnosis-based clinical decision rule and was the principal author of the manuscript.

ELH was responsible for help with design and presentation and with conceptualization of the presented research strategy.

## Pre-publication history

The pre-publication history for this paper can be accessed here:


